# Severe class ii malocclusion in a growing patient treated with functional orthopedics and fixed appliances

**DOI:** 10.21142/2523-2754-1403-2026-305

**Published:** 2026-07-10

**Authors:** Herbert Orrego, Erika Buendía

**Affiliations:** 1 Faculty of Dentistry, Postgraduate Program in Orthodontics, Universidad Nacional Mayor de San Marcos. Lima, Peru. herbertorrego@hotmail.com Universidad Nacional Mayor de San Marcos Faculty of Dentistry Postgraduate Program in Orthodontics Universidad Nacional Mayor de San Marcos Lima Peru herbertorrego@hotmail.com; 2 Faculty of Dentistry, Orthodontics Postgraduate Resident, Universidad Nacional Mayor de San Marcos. Lima, Peru. cd.erikabuendia@gmail.com Universidad Nacional Mayor de San Marcos Faculty of Dentistry Orthodontics Postgraduate Resident Universidad Nacional Mayor de San Marcos Lima Peru cd.erikabuendia@gmail.com

**Keywords:** class II malocclusion, functional appliances, case report, interceptive and corrective orthodontics, maloclusión de clase II, aparatos funcionales, informe de caso, ortodoncia interceptiva-correctiva

## Abstract

This report presents the clinical management of a growing patient with severe Class II Division 1 malocclusion treated using a two-phase approach. The initial phase consisted of functional orthopedic therapy with an open Klammt elastic activator, followed by a second phase using fixed orthodontic appliances. The advantages, limitations, and effects associated with this treatment strategy are discussed. In this case, a 17-mm overjet was successfully reduced, adequate occlusal relationships were established, the existing lip interposition habit was eliminated, mandibular functional movements free of interferences were achieved, and a marked improvement in facial soft-tissue esthetics was obtained.

## RESUMEN

Se presenta el caso clínico de un paciente en crecimiento portador de una severa maloclusión de clase II división 1 tratado en dos fases: una inicial, en la que se empleó un activador elástico abierto de Klammt, y una posterior, en la que se usó aparatología fija. Se analizan las ventajas, desventajas y efectos que este abordaje genera. En el presente caso, se pudo corregir un resalte horizontal de 17 mm, conseguir adecuadas relaciones oclusales, eliminar el hábito de interposición labial existente, obtener movimientos funcionales mandibulares libres de interferencias y conseguir una mejora estética en los tejidos blandos.

## INTRODUCTION

Although Class I malocclusions are the most prevalent in the general population ^1^^,^^2^, Class II malocclusions account for the majority of orthodontic consultations ^3^^,^^4^. This is largely due to the associated esthetic and functional impairments, which are more readily perceived by patients and their parents.

Class II malocclusion is characterized by a skeletal discrepancy resulting from mandibular retrusion or underdevelopment, maxillary prognathism or enlargement, or a combination of these factors. Its etiology is multifactorial, involving hereditary, environmental, and functional components.

Deleterious oral habits such as lip interposition, tongue thrusting, and oral breathing are frequently associated and may exacerbate the condition.

Depending on severity, treatment options include orthopedic, orthodontic, surgical, or combined approaches. The indication of two-phase treatment protocols remains controversial. Advocates cite benefits such as etiologic intervention, habit control, prevention of dental trauma, early esthetic and functional improvement, potential long-term stability, and positive psychosocial effects ^5^^,^^6^.

Critics argue that two-phase therapy increases treatment duration, cost, and complexity without demonstrating superior outcomes compared to single-phase treatment ^7^^,^^8^.

The aim of this article is to present a case of Class II Division 1 malocclusion managed initially with functional orthopedic therapy and subsequently completed with fixed orthodontic appliances.

## CASE PRESENTATION

A 12-year-old male patient sought orthodontic treatment at the postgraduate clinic of the Universidad Nacional Mayor de San Marcos. The chief complaint, expressed by both the patient and his parents, was: “I want to fix my teeth.” Medical history included adenoid hypertrophy and allergic rhinitis.

**Figure 1:**
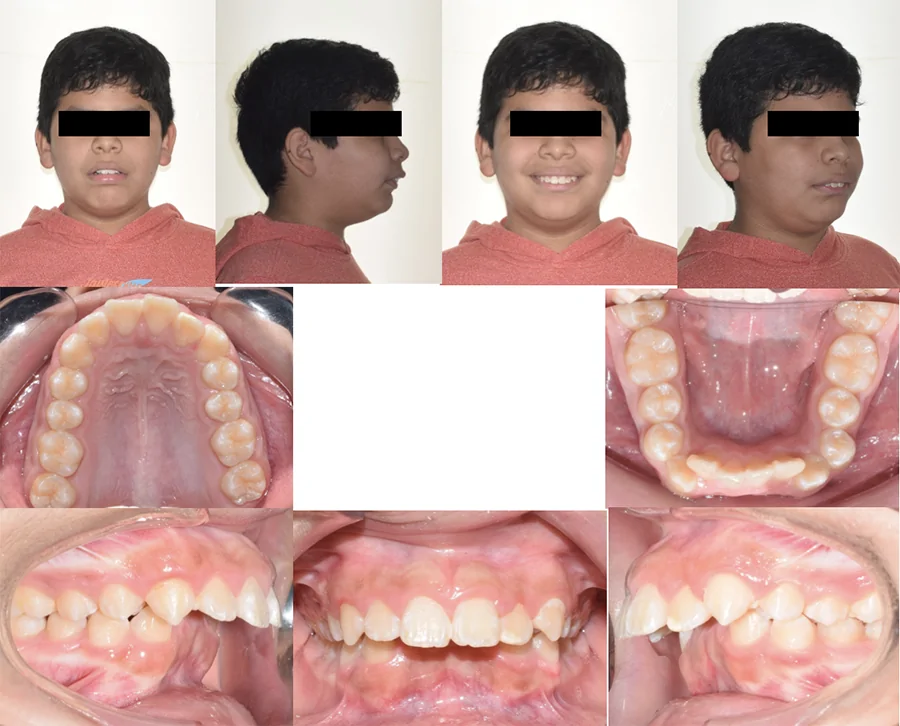
Initial extraoral and intraoral photographs

Extraoral examination revealed a symmetrical mesofacial patient with a robust build, lip incompetence, disproportionate facial thirds, a convex profile, protrusive upper lip, retrusive lower lip and chin, mixed breathing with oral predominance, and lip interposition. Intraorally, the patient presented with permanent dentition, square-shaped arches, bilateral Class II molar and canine relationships, a 17-mm overjet, 100% overbite, mild maxillary crowding, severe mandibular crowding, a pronounced curve of Spee, and a 1-mm maxillary midline deviation to the right. The panoramic radiograph showed findings consistent with chronological age and normal osseous structures (Figure 1).

**Figure 2:**
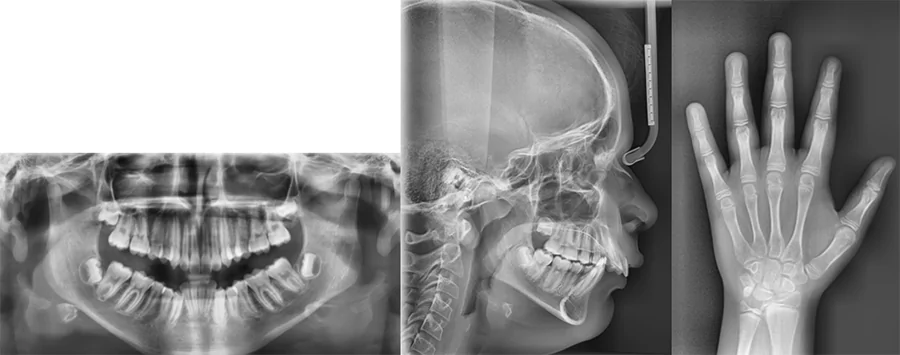
Pretreatment panoramic, cephalometric, and hand-wrist radiographs

The lateral cephalometric radiograph demonstrated a skeletal Class II relationship due to mandibular retrusion and maxillary protrusion, a hyperdivergent mandibular plane, proclined maxillary incisors, retroclined mandibular incisors, and a hyperdivergent growth pattern. The hand- wrist radiograph revealed the presence of the sesamoid bone, indicating the onset of the pubertal growth spurt (Figure 2).

### Treatment objectives

Treatment objectives included improvement of the skeletal Class II relationship, correction of incisor inclination, reduction of overjet and overbite, elimination of the lip interposition habit, correction of canine and molar relationships, normalization of the curve of Spee, achievement of optimal dental interdigitation, improvement of the facial profile, enhancement of the respiratory pattern, and establishment of an adequate lip seal.

### Treatment alternatives

Three treatment options were considered: a single-phase approach involving maxillary distalization combined with mandibular advancement and fixed appliances; extraction of maxillary premolars followed by fixed appliances, maintaining Class II molar and Class I canine relationships; and a two-phase approach consisting of functional orthopedic therapy followed by fixed orthodontic treatment.

After discussion of the alternatives, the patient’s parents selected the two-phase treatment option.

### Treatment progress

An open elastic Klammt activator was fabricated following a constructive bite registration with approximately 7 mm of mandibular advancement. Interocclusal acrylic was incorporated to guide dental eruption. Monthly follow-up visits included appliance activation and selective acrylic reduction.

**Figure 3:**
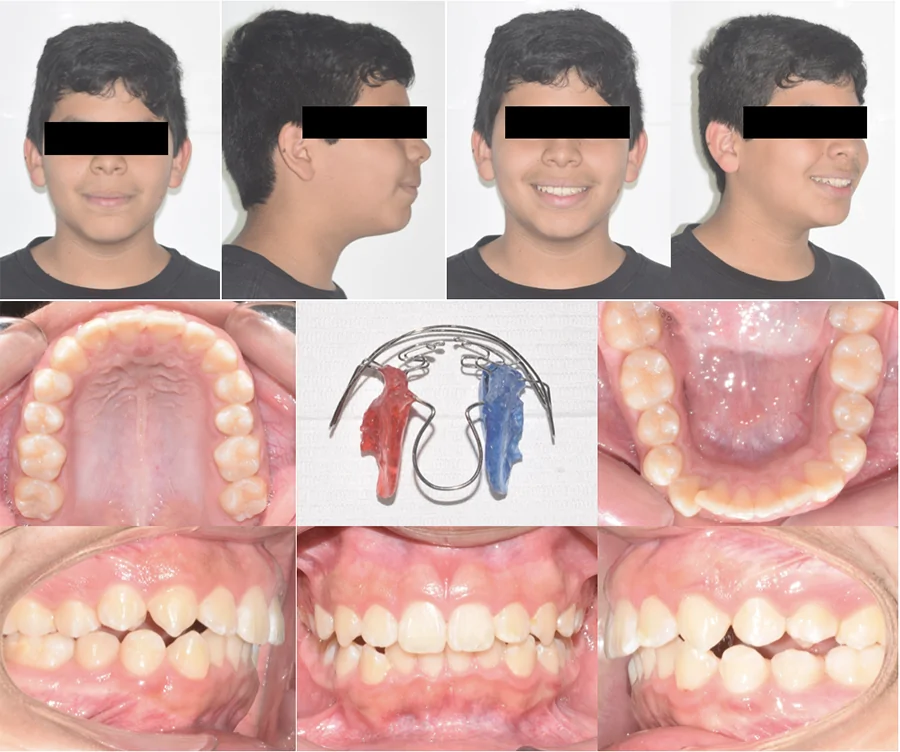
Photographs after 14 months of open elastic activator therapy (central photo). Note facial and occlusal changes.

**Figure 4:**
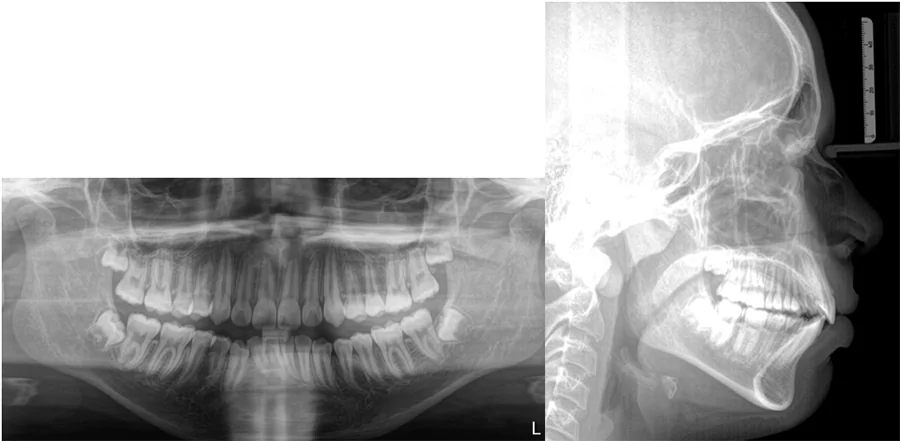
Control radiographs at the end of the functional orthopedic phase

After 14 months, bilateral Class I molar relationships were achieved, overjet was reduced to 3 mm, overbite to 30%, the curve of Spee improved, lip competence was established, the respiratory pattern improved, and a marked enhancement of the facial profile was observed (Figure 3). Residual objectives included resolution of mild mandibular crowding, final dental interdigitation, and interference-free mandibular excursions. Radiographic changes are shown in Figure 4.

The second phase involved placement of preadjusted fixed appliances with a 0.022-inch slot. Following alignment and leveling, finishing procedures were performed using 0.019 × 0.025-inch stainless-steel archwires, Class II and triangular intermaxillary elastics, and final settling with braided stainless-steel archwires.

**Figure 5:**
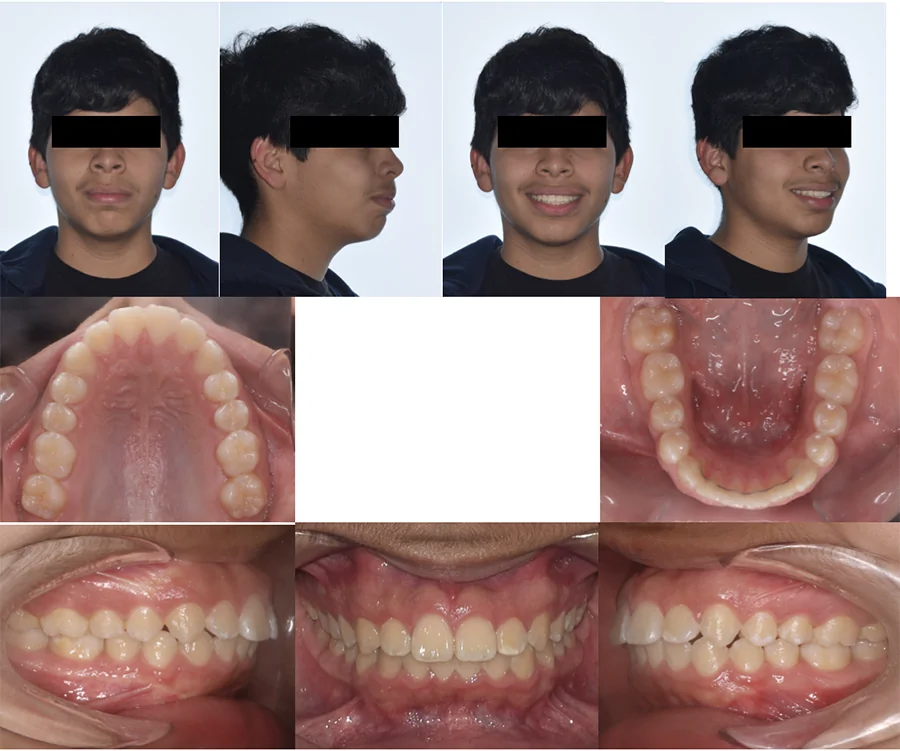
Post-treatment photographs

**Figure 6:**
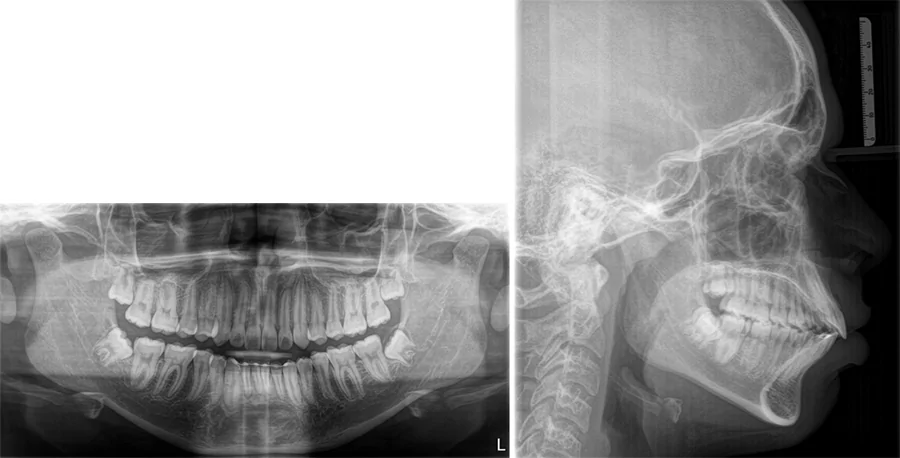
Control radiographs at the end of treatment

At completion, bilateral Class I molar and canine relationships were obtained, overjet measured 2 mm, overbite 30%, the lip interposition habit was eliminated, facial profile improved substantially, lip competence was achieved, and mandibular movements were free of interferences (Figures 5 and 6 ).

Total treatment duration was 32 months (14 months orthopedic phase, 18 months fixed appliance phase). Retention consisted of a bonded mandibular canine-to-canine retainer and a removable maxillary circumferential retainer.

Cephalometric values (Table 1) demonstrate an improvement in the maxillomandibular relationship, changes in incisor inclination, and improved lip position. Regional superimpositions reveal chin projection (4 mm), chin downward displacement (5 mm), maxillary molar extrusion (3 mm), downward displacement of point A (3 mm), slight clockwise rotation of the palatal plane (2°), proclination of the mandibular incisors (23°), retrusion and intrusion of the maxillary incisors (1 mm each), retrusion of the upper lip, and protrusion of the lower lip.

**Table 1:** Pretreatment and posttreatment cephalometric values

Measurements	Pretreatment	Posttreatment
SNA (°)	87	83
SNB (°)	76	78
ANB (°)	11	5
Wits appraisal (mm)	5.5	2.5
Maxillomandibular angle (°)	29	26
FMA (°)	30	26
MP-SN (°)	34	39
U1 / Palatal plane (°)	120	122
U1 / A-Pog (mm)	16	6.5
IMPA (°)	126	103
L1 / A-Pog (mm)	0	9
Upper lip / E-plane (mm)	5	2
Lower lip / E-plane (mm)	0	5

**Figure 7:**
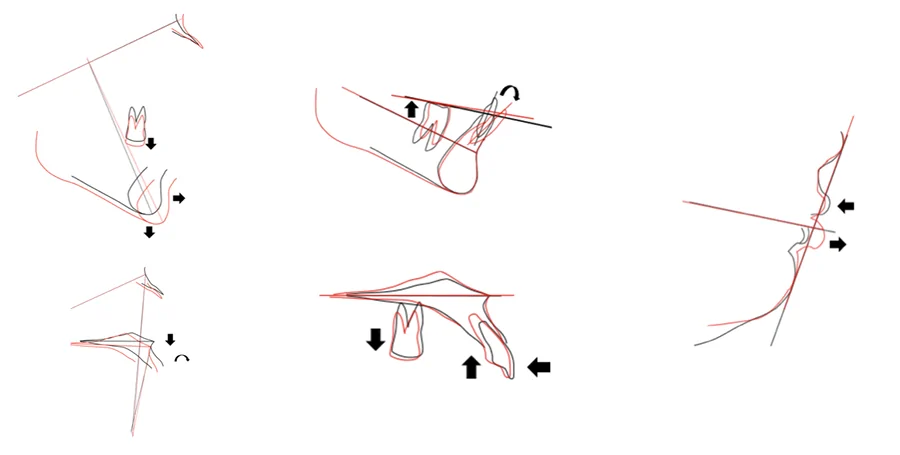
Regional superimpositions. Black: pretreatment; red: posttreatment.

## DISCUSSION

Classical authors such as Proffit ^9^ and Graber ^10^ advocate combining functional orthopedic appliances with fixed orthodontic appliances for the treatment of Class II malocclusions in growing patients. This approach considers not only the skeletal effects of functional orthopedics on the position and growth of the basal bones, but also the marked dentoalveolar effects produced by this therapy-such as overjet and overbite reduction, changes in incisor inclination, extrusion of posterior teeth, and improvement in arch form ^11^^,^^12^.

In the present case, the lip interposition habit was corrected during the constructive bite registration for fabrication of the Klammt activator, as mandibular advancement eliminated the space required for the lip habit. The effect on the facial profile was also remarkable, with a significant reduction in facial convexity. Effects on soft tissues have likewise been reported in the literature.

By improving the maxillomandibular relationship and dentoalveolar conditions through functional therapy, the subsequent orthodontic phase becomes shorter and simpler, which under a utilitarian rationale should reduce overall treatment cost.

In this case, an overjet reduction from 17 mm to 3 mm was achieved in 14 months, along with the previously described effects. Additionally, correction of the maxillomandibular relationship facilitated fixed appliance therapy. The literature reports adequate stability of this treatment scheme, particularly in sagittal correction ^13^.

An often underemphasized aspect is that early overjet correction significantly reduces the risk of traumatic dental injuries.

We believe that achieving improvements in esthetic, dental, and functional aspects constitutes delivery of high-quality care to our patients. In this case, combining functional orthopedic therapy with fixed appliances allowed us to achieve these objectives. Management of growing patients requires careful case selection; patient cooperation is essential, growth pattern analysis strongly influences prognosis, and family commitment is crucial when treating pediatric patients.

## CONCLUSIONS

A growing patient with skeletal Class II malocclusion, severe overjet, and mandibular retrognathia was successfully treated using an open elastic Klammt activator followed by fixed orthodontic appliances. Normal static and dynamic occlusal relationships were achieved, and facial profile esthetics improved significantly. Favorable outcomes can be expected when appropriate case selection is made, and adequate patient cooperation is obtained.

## Sources of financing:

Nill

## References

[1] Al-Hammad NS, Al-Hussainan TS, Al-Jawad MA (2025). The prevalence of orthodontic malocclusion in children aged 10-12 years an epidemiological study. BMC Oral Health.

[2] Alhammadi MS, Al-Khalifa KS, Halboub E, Fayed MS, Almuzian M, El-Sheikh M (2018). Global distribution of malocclusion traits: a systematic review. Dental Press J Orthod.

[3] Ardani IGAW, Aini H, Narmada IB, Deshmukh S, Nugraha AP (2023). Class II Division 1 malocclusion treatment trends in the last 10 years by skeletal classification a review article. J Int Oral Health.

[4] Giuntini V, McNamara JA, Franchi L (2023). Treatment of Class II malocclusion in the growing patient early or late?. Semin Orthod.

[5] OBrien K, Wright J, Conboy F, Appelbe P, Davies L, Connolly I (2009). Early treatment for Class II Division 1 malocclusion with the Twin-block appliance a multicentre randomized controlled trial-long-term follow-up. Am J Orthod Dentofacial Orthop.

[6] Thiruvenkatachari B, Harrison J, Worthington H, OBrien K (2015). Early orthodontic treatment for Class II malocclusion reduces the chance of incisal trauma results of a Cochrane systematic review. Am J Orthod Dentofacial Orthop.

[7] Kumar M, Nongthombam H (2025). Debunking the two-phase treatment myth. J Indian Orthod Soc.

[8] Veitz-Keenan A, Liu N (2019). One-phase or two-phase orthodontic treatment for Class II Division 1 malocclusion. Evid Based Dent.

[9] Proffit WR, Fields HW, Larson BE, Sarver DM (2019). Contemporary Orthodontics.

[10] Graber LW, Vanarsdall RL, Vig KWL, Huang GJ (2016). Orthodontics: Current Principles and Techniques.

[11] Malta LA, Baccetti T, Franchi L, Faltin K, McNamara JA (2010). Long-term dentoskeletal effects and facial profile changes induced by bionator therapy. Angle Orthod.

[12] Franchi L, Pavoni C, Faltin K, McNamara JA, Cozza P (2013). Long-term skeletal and dental effects and treatment timing for functional appliances in Class II malocclusion. Angle Orthod.

[13] Pavoni C, Cretella Lombardo E, Lione R, Faltin K, McNamara JA, Cozza P (2018). Treatment timing for functional jaw orthopaedics followed by fixed appliances a controlled long-term study. Eur J Orthod.

